# Primary Biliary Cholangitis in a Patient With Multiple Sclerosis: A Case Report

**DOI:** 10.7759/cureus.79162

**Published:** 2025-02-17

**Authors:** Thaer K Swaid, Omar Galal, Ahmad Al Rifai

**Affiliations:** 1 Gastroenterology and Hepatology, Sheikh Shakhbout Medical City, Abu Dhabi, ARE; 2 Medicine, Khalifa University, Abu Dhabi, ARE

**Keywords:** autoimmune disorder, coexisting diseases, immune system dysregulation, multiple sclerosis, primary sclerosing cholangitis (psc)

## Abstract

Multiple sclerosis (MS) is a chronic autoimmune condition characterized by central nervous system demyelination. Its coexistence with primary biliary cholangitis (PBC), another autoimmune disease, is rarely described in the literature. This is the case of a 35-year-old Emirati female patient with a history of PBC who presented with unsteady gait and neurological symptoms. Neurological examination revealed dysmetria, wide-based gait, and brisk reflexes. MRI findings confirmed MS, and she was treated with methylprednisolone and disease-modifying therapies. MS and PBC share immune dysregulation, involving genetic loci like CD28 and T-cell-mediated mechanisms. Their coexistence complicates diagnosis due to overlapping symptoms such as fatigue and neurological impairment. While rare, concurrent autoimmune diseases in MS patients may influence disease progression and disability outcomes. This case emphasizes the need for multidisciplinary care in managing coexisting autoimmune diseases and for further research regarding the shared mechanisms of these coexisting autoimmune diseases.

## Introduction

Multiple sclerosis (MS) is a chronic inflammatory disease characterized by the demyelination of the white matter of the brain and spinal cord. It is a common cause of chronic neurological disability in young adults in developed countries [1]. MS patients have a higher risk of developing additional autoimmune disorders compared to the general population. These include uveitis, inflammatory bowel disease (IBD), Bell's palsy, Guillain-Barré syndrome, bullous pemphigoid, type 1 diabetes, ulcerative colitis, and autoimmune thyroiditis [2]. The likelihood of concurrence between MS and autoimmune diseases varies among individuals and is due to several genetic, humoral, or environmental factors [3].

Primary biliary cholangitis (PBC) is a rare autoimmune disease that primarily attacks bile ducts. The deterioration begins as inflammation and scarring that can rapidly progress to cholestatic disease and liver cirrhosis [4,5]. PBC affects women more than men in a ratio of 9:1 and is most frequently diagnosed in people 40 years or older [6]. While often asymptomatic at diagnosis, patients may later experience fatigue and pruritus. The coexistence of MS and PBC is rare, with few reported cases in the literature. Research suggests that immune dysregulation and shared genetic predispositions may contribute to the coexistence of these diseases [7,8]. This report presents a case of newly diagnosed MS in a middle-aged woman with pre-existing PBC.

## Case presentation

The patient is a 35-year-old Emirati woman who presented with an unprecedented unsteady gait for approximately 20 days. The symptoms remained unchanged since onset. Additionally, she recalled experiencing numbness in her right hand one year earlier, which resolved spontaneously. She denied any history of trauma, accidents, weakness, headaches, or bladder or bowel dysfunction.

**Figure 1:**
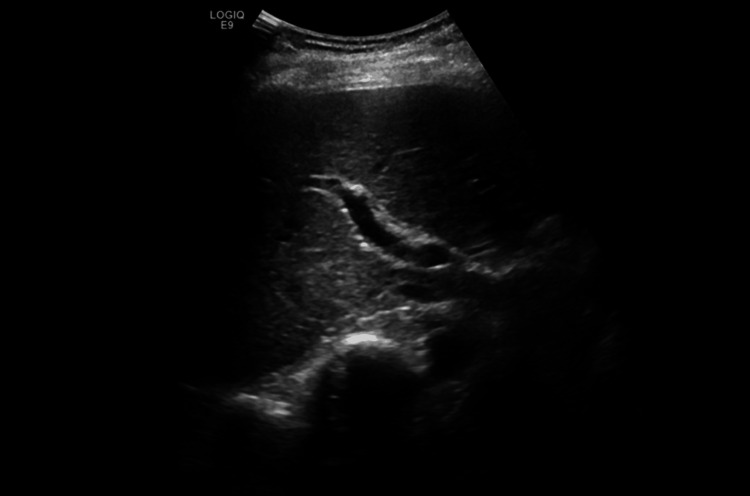
Ultrasound of the liver shows normal parenchyma and no biliary duct dilation

Her past medical history is significant for a metallic heart valve, which was placed following a diagnosis of endocarditis. She is also known to have PBC, diagnosed approximately one year earlier after presenting with fatigue and mild pruritus. Investigations revealed positive antimitochondrial antibodies (AMAs), while M2 positivity was not tested. An ultrasound at the time was unremarkable, showing a normal biliary system without evidence of intra- or extrahepatic ductal dilation (Figure 1). She was started on ursodeoxycholic acid (500 mg, orally, twice a day) and has since maintained stable liver enzyme levels without hepatic complications. At the time of PBC diagnosis, her liver enzyme levels were alkaline phosphatase (ALP): 449 IU/L, gamma-glutamyl transferase (GGT): 680 IU/L, aspartate aminotransferase (AST): 54 IU/L, and alanine transaminase (ALT): 133 IU/L. Upon presentation, there was no evidence of chronic liver disease, and the elevated liver enzymes were an incidental finding. A liver biopsy was not performed.

On admission, a neurological examination revealed that the patient was alert and oriented. All cranial nerves were grossly intact. Mild dysmetria was noted on finger-to-nose testing in both upper extremities, and Romberg’s sign was positive. The patient’s gait was wide-based. Muscle strength was 5/5 in the bilateral upper extremities and 4/5 in the left lower extremity hip flexor. Reflexes were brisk in both the upper and lower extremities. She also exhibited ocular symptoms, including left-eye optic neuritis.

**Figure 2:**
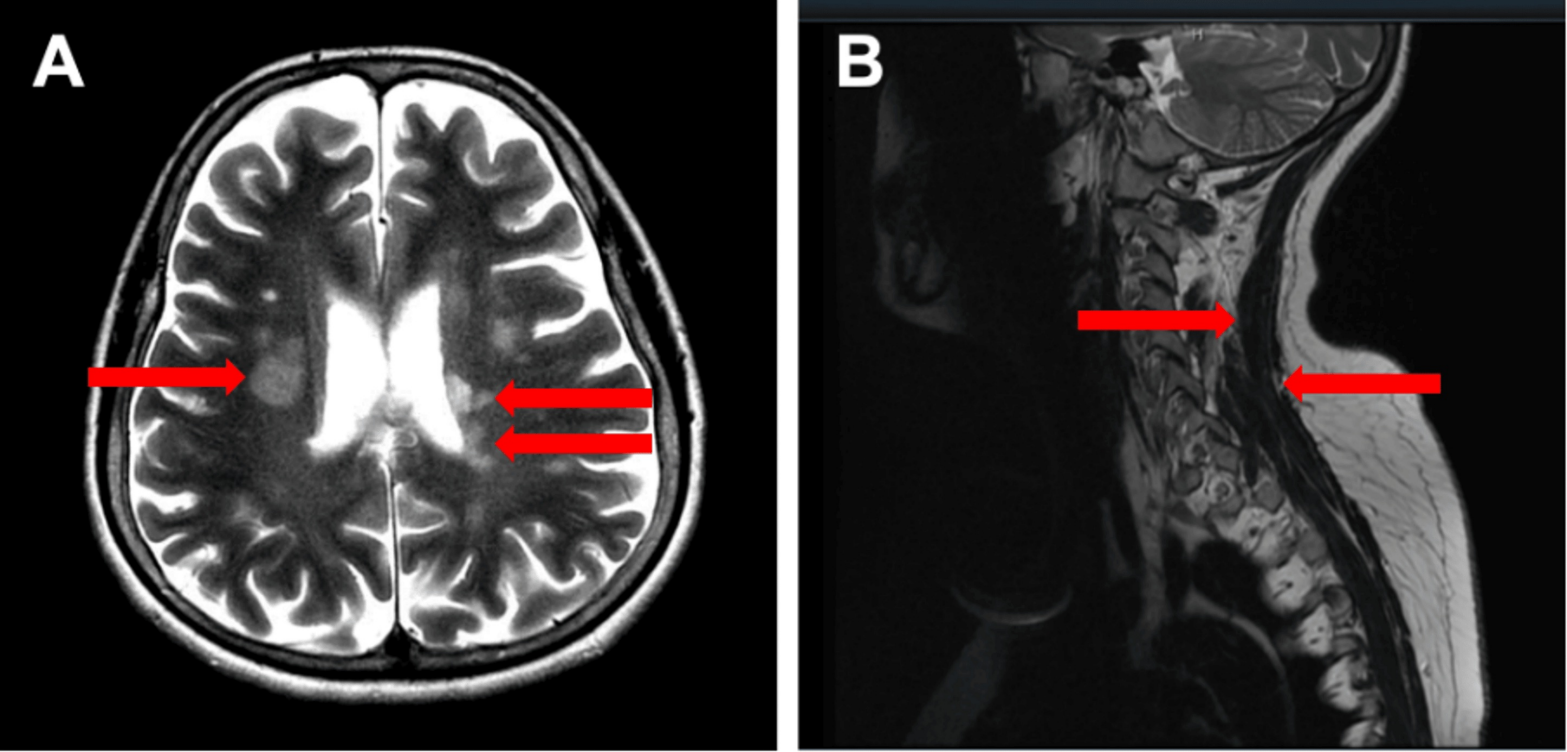
(A) T2-weighted axial MRI of the brain shows hyperintense periventricular lesions (red arrows). (B) T1-weighted sagittal MRI of the cervical and thoracic spine shows hyperintense lesions (red arrows)

Due to worsening unsteady gait, an MRI of the brain and cervical spine was performed, revealing multiple periventricular white matter lesions consistent with a diagnosis of MS (Figure 2) [6]. The patient underwent an extensive autoimmune and connective tissue disease workup, which was negative. Aquaporin-4 (AQP4) antibody testing was not performed based on MRI findings and neurology clinical judgment. CSF testing was also not conducted. She was treated with methylprednisolone (intravenously, 1 g/day), leading to symptomatic improvement. She was subsequently started on disease-modifying therapies, including Gilenya (0.5 mg, orally, daily) and Fampyra (10 mg, orally, twice a day).

During a two-year follow-up, the patient experienced two MS relapses despite ongoing treatment. These relapses presented with unsteady gait, severe dizziness, and repeated vomiting and were managed with pulse steroids (IV methylprednisolone 1 g/day). A repeat MRI of the brain showed a new right frontal lobe plaque without enhancement or activity, while a repeat cervical spine MRI revealed lesion progression and new lesions, indicating disease advancement.

## Discussion

This case underscores the prevalence of coexisting autoimmune diseases, namely, PBC and MS. Among autoimmune diseases associated with MS, PBC is extremely rare and accounts for approximately 1.4% of MS patients [7]. In fact, there are only a handful of such documented cases in the current literature [8-10].

While the concurrence of MS and PBC is rarely described in the literature, a case report by Pontecorvo et al. in 1992 is one of the first to shed light on the possible connection between these two diseases. Since then, a handful of other case reports have supported this association. A case series described three women (aged 27, 51, and 52) with coexisting MS and PBC. The 52-year-old developed MS three years after a PBC diagnosis, responding initially to treatment but experiencing relapses within a year. The 27-year-old presented with severe neurological symptoms and was diagnosed with MS, followed by PBC two months later; she succumbed to complications within a year. The 51-year-old, with a history of uveitis and Behcet’s disease, developed PBC eight years earlier and later presented with vertigo and paresthesia, with a brain MRI confirming MS [9].

Previous case reports have described similar presentations, highlighting that the presence of fatigue, neurological symptoms, and mild hepatic dysfunction can complicate the diagnostic process [8-10]. Unlike typical PBC cases that progressively worsen, this patient’s liver function remained stable, suggesting that close monitoring is crucial in determining disease progression. Moreover, her MS followed an expected relapsing-remitting course, with exacerbations requiring pulse steroid therapy.

A unique feature of this case was the presence of optic neuritis, which is a well-recognized symptom of MS but is not typically seen in PBC [5]. This highlights the need for thorough neurological evaluations in patients with autoimmune liver diseases who develop unexplained neurological symptoms. Additionally, the patient’s stable liver function at diagnosis suggests that early detection of overlapping autoimmune conditions may help optimize treatment and prevent complications. Despite ongoing treatment, the patient experienced disease progression on imaging. This aligns with findings from other case reports indicating that concurrent autoimmune diseases do not necessarily alter the relapse rate in MS but may modify overall disease progression [7].

The immunopathology and mechanisms between MS and PBC can help explain the connection between these two diseases. These two diseases are characterized by immune dysregulation and chronic inflammation, potentially sharing genetic susceptibility, humoral pathogenesis, and environmental triggers [11]. Both MS and PBC involve dysregulation of T-cell-mediated immunity. In MS, CD4+ and CD8+ T cells target myelin, while in PBC, autoreactive T cells attack biliary epithelial cells [12]. It is also postulated that the high levels of AMAs seen in up to 95% of cases of PBC and suggesting a loss of immune tolerance are similar to the mechanisms that lead to loss of immune tolerance in MS [13]. Another theory involves a potential genetic association between the two disease processes, namely, shared loci such as CD28, associated with susceptibility to autoimmune conditions including MS and PBC, suggesting a potential common immunological basis [14,15].

The clinical implications of the coexistence of MS and PBC relate to the challenges of distinguishing between the often similar and overlapping symptoms, such as fatigue and neurological impairment, which are present in both diseases [15,16]. While MS and PBC typically progress individually, the concurrence of both diseases can modify the course of the disease progression. Interestingly, a study involving 1,700 MS patients showed that patients with MS and another concurrent autoimmune disease have considerably fewer disabilities (according to the Expanded Disability Status Scale) compared to patients with MS alone [7]. While the mechanism is not currently clear, one possible explanation is that exposure to more antigens can lead to immune tolerance and a milder disease progression. Additionally, the relapse rate appears to be unaffected by the concurrence of MS with autoimmune diseases, PBC included [7].

## Conclusions

This case illustrates the uncommon concurrence of MS and PBC, both of which are autoimmune disorders marked by immune system dysfunction and persistent inflammation. The presence of overlapping symptoms poses challenges to accurate diagnosis and effective management, highlighting the necessity for a collaborative, multidisciplinary approach.

This case underscores the need for heightened awareness of secondary autoimmune diseases in patients with PBC and the importance of proactive screening. Clinicians should remain vigilant when such patients present with seemingly mild symptoms, as they may indicate an underlying autoimmune process. Future research should aim to clarify the genetic and immunological links between MS and PBC while refining screening strategies and optimizing multidisciplinary care to improve patient outcomes.
